# Test–retest reliability of putting-related variables in medium-to-high handicap golf players

**DOI:** 10.1038/s41598-024-62183-z

**Published:** 2024-05-20

**Authors:** Xavier García-Massó, Israel Villarrasa-Sapiña, Nuria Ortega-Benavent, Sergio Montalt-García, José L. Toca-Herrera

**Affiliations:** 1 Departamento de Expresión Musical, Plástica y Corporal Universidad de Valencia, Valencia, Spain; 2 Human Movement Analysis Group (Human), Universidad de Valencia, Valencia, Spain; 3 Departamento de Educación Física y Deportiva, Universidad de Valencia, Valencia, Spain; 4 Institute of Biophysics, Department of Bionanosciences, University of Natural Resources and Life Sciences Vienna (BOKU), Muthgasse 11, 1190 Vienna, Austria

**Keywords:** Reliability, Putting, Performance assessment, Accelerations, Motor control, Health care

## Abstract

This manuscript aims to study the reliability of different variables related to performance and acceleration during the golf putt in players with medium-to-high handicaps and to determine the number of attempts necessary to find reliable values for these variables. Eight males and two females [55.67 (13.64) years, 78.4 (11.4) kg, 1.75 (7.95) m] participated in two experimental sessions separated by one week. In these sessions, they performed three blocks of 10 putts trying to stop the golf ball at the center of a dartboard painted 2 m away. The performance was assessed depending on the area of the dartboard where the ball stopped, and the acceleration signals were acquired using the Xsens Dot. The results showed that to evaluate performance, 18 trials were necessary to reach reliable values using the 0–10 scoring system, and 28 trials were necessary for the 0–3 scoring system. Regarding the reliability of the accelerometer-related variables, 7 attempts were necessary to obtain good-to-excellent reliability values for most of the variables. It could be concluded that putting in medium-to-high handicap golf players can be reliably measured using the abovementioned protocol.

## Introduction

Putting is the most performed shot on a golf course. On average, in a 72-handicap course, 36 shots are expected to be putts. Specifically, Pelz reported that 43% (± 2%) of the shoots of a course are putts^1^, and several studies have shown a positive relationship between putting performance and overall course score^2–4^. Putting ability is the most important determinant of the earnings of PGA golfers^5^.

Moreover, putting skills can be developed by everyone, as their movement requires only the coordination of a few joints and does not require specialized training. This stands in contrast to other golfing techniques, such as drive swinging, which can be very difficult for people who never play golf due to the intricate coordination of numerous joints involved in the movement^6^. For this reason, putting is one of the most commonly used skills in motor learning research both for golf (independently of the levels) and for nongolf players^7–14^.

In most of these studies, putting performance has been measured as the distance from the stop position of the ball to a target or hole. For that purpose, two main methods have been used: radial error^13^ or a dartboard score around the target^11^. However, published studies have not coincided with the number of shots completed in the experimental protocols. Thus, studies in which 5 to 10 trials were performed^11–13^ until studies that used protocols of 40 to 50 trials were found^7,14^.

This is important because the measurement protocols used to evaluate putting performance should be reliable, and the number of shots included in the protocols could be a key factor in determining reliability^15,16^. Nevertheless, none of the published manuscripts in which putting performance was measured reported test–retest reliability values depending on the number of shots performed. Schweizer et al. conducted a reliability study of putt performance, but their protocol involved a single session to determine the stability of the performance measurements^17^. Using the Spearman-Brown formula, they established that 14 trials were not enough to consider the distance between the ball and the hole as a performance variable (ICC = 0.71; they established good reliability at 0.8).

Furthermore, different factors affecting putting performance, such as biomechanical^18^ or physiological factors^19^, have also been measured. From a biomechanical point of view, several studies have provided evidence of the reliability of different swing golf variables. Richardson et al. proposed a method to determine the impact point during the swinging of golf putt, and they reported excellent reliability^20^. Mackenzie and Evans developed a system based on light-emitting diodes and high-speed cameras to capture the head motion of a putter and demonstrated its validity and reliability in measuring the face angle, stroke path, putter speed, and impact spot at the moment of ball contact^21^. Nevertheless, in recent years, inertial measurement units (IMUs) have gained special attention in sport-related biomechanical analysis since they are ubiquitous, inexpensive, require minimal user intervention, and can be used in representative training environments^22^. In this sense, Jensen et al. developed an IMU-based system to detect golf putts and provide real-time feedback^23^. In this study, they proposed interesting parameters of golf putting computed using only an IMU sensor attached to the head of the putter. However, there is no information about the test–retest reliability of these variables.

Together with the increase in the number of amateur golf players in recent years, these findings encourage more studies to be carried out to provide reliable procedures to measure performance and biomechanics in this population^25^. It should also be taken into account that the number of shots required to measure performance and biomechanics variables could be greater than those used in some experimental studies (from 5 to 10 as described above) because medium–high handicap players have great shot-by-shot variability^24^, and therefore, they could require more putts to reach good reliability values^16^. This article aims to fill this gap in knowledge since there is a need for experimental data about the reliability of putting performance and biomechanical variables depending on the number of shots used during the evaluation since there is no scientific evidence to date. These data could be of interest both for researchers involved in measuring these variables in a reliable way and for practitioners who can use the protocols derived from this study to measure putting performance (and biomechanics variables as well) in their pupils to check the effect of the learning interventions that they provide. Therefore, the objective of the present study was to determine the test–retest reliability of performance and accelerometer-related variables during golf putting in medium-to-high handicap players. This aim also implies determining the number of attempts needed to reach reliable values for these variables. We hypothesize that variables derived from both biomechanical and performance aspects can be reliably quantified with a maximum of 30 trials.

## Results

**Figure 1:**
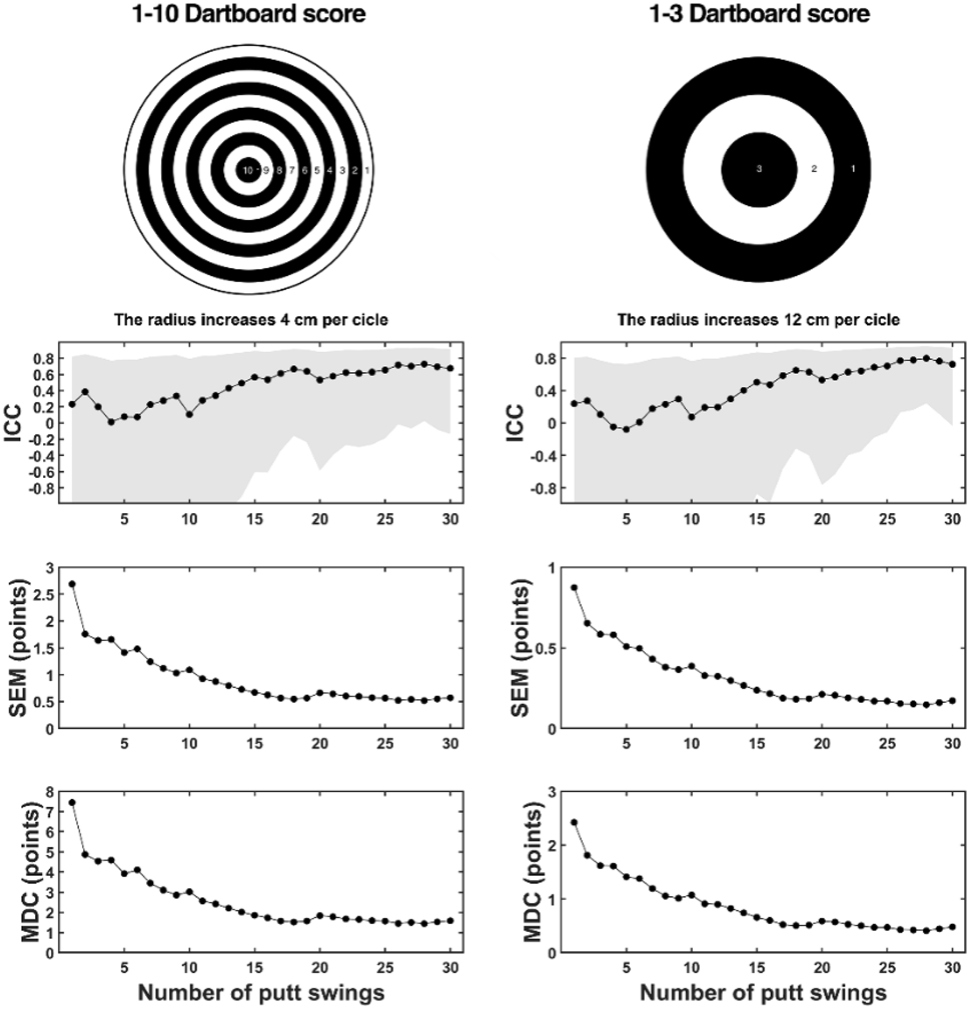
The ICC, SEM and MDC parameters for each dashboard score as a function of the number of trials used to compute performance. The shadow of the ICC layers represents the 95% confidence interval. ICC: intraclass correlation coefficient; SEM: standard error of the measurement and MDC: minimum detectable change.

Figure 1 shows the reliability parameters for the putt performance score depending on the number of trials performed and the width of the target zones. The ICCs increased from trials 1 to 28 for both targets, while the SEMs and MDCs decreased as the number of trials increased. The RTI established that for the case of 0–10 dartboard scores, 17–18 trials are sufficient (Table 1), which coincides with the point at which the ICC, SEM and MDC start to stabilize (Fig. 1). In contrast, for the 0–3 dartboard score, the RTI established 28 trials for the ICC (although 17 for the SEM and MDC). As seen in the ICC layer for the 0–3 dartboard score, the value stabilized at 28 trials instead of at 18 trials, as has been found for the 1–10 dartboard score but at the highest reliability level (i.e., 0.8 instead of 0.67).

**Table 1:** Required number of shots (per session) for the calculation of each variable depending on the reliability index used.

Variable	ICC	SEM	MDC
Score (0–10)	18	17	17
Score (0–3)	28	17	17
Putt time (s)	5	4	4
Backswing time (s)	3	2	2
Downswing time (s)	1	4	4
Follow-through time (s)	2	5	5
Swing time (s)	1	3	3
BS/DS time ratio	3	6	6
DS/FT time ratio	1	6	6
Backswing peak (m s^−2^)	1	5	5
Downswing peak (m s^−2^)	1	7	7
Backswing peak time (s)	2	2	2
Downswing peak time (s)	1	3	3
Total resultant velocity (m s^−1^)	1	4	4
Backswing resultant velocity (m s^−1^)	1	4	4
Downswing resultant velocity (m s^−1^)	1	3	3

ICC: intraclass correlation coefficient; SEM: standard error of the measurement; MDC: minimum detectable change; BS: backswing; DS: downswing; FT: follow-through.

Tables 2, 3 and 4 show that the reliability of some of the accelerometer variables did not reach acceptable levels. For example, the ICCs for follow-through time, backswing peak time and downswing impulse did not reach 0.5 regardless of the number of putts performed. For the other parameters, good-to-excellent reliability values were found. In Table 1, the number of trials required to reach reliable accelerometer-related variables (i.e., using the RTI) is reported. Seven trials were sufficient to measure most of the variables reliably, which contrasts with the 18 trials or 28 trials needed to measure the performance score.

**Table 2:** The intraclass correlation coefficient for accelerometer-related variables during putting.

	Number of trials						
	1	5	10	15	20	25	30
Putt time (s)	0.19 [− 2.79 to 0.81]	0.71 [− 0.04 to 0.93]	0.71 [− 0.18 to 0.93]	0.65 [− 0.27 to 0.91]	0.65 [− 0.25 to 0.91]	0.69 [− 0.19 to 0.92]	0.68 [− 0.22 to 0.92]
Backswing time (s)	0.24 [− 2.40 to 0.82]	0.92 [0.69 to 0.98]	0.82 [0.23 to 0.95]	0.73 [0.00 to 0.93]	0.68 [− 0.11 to 0.92]	0.65 [− 0.20 to 0.91]	0.75 [0.06 to 0.94]
Downswing time (s)	0.77 [0.16 to 0.94]	0.77 [0.12 to 0.94]	0.85 [0.37 to 0.96]	0.79 [0.20 to 0.95]	0.79 [0.23 to 0.95]	0.80 [0.27 to 0.95]	0.81 [0.31 to 0.95]
Follow-through time (s)	− 2.73 [− 230.05 to 0.25]	− 0.42 [− 6.59 to 0.67]	− 0.11 [− 2.03 to 0.69]	0.02 [− 1.82 to 0.73]	− 0.05 [− 1.77 to 0.70]	0.18 [− 1.08 to 0.76]	0.00 [− 1.22 to 0.69]
Swing time (s)	0.84 [0.37 to 0.96]	0.90 [0.45 to 0.98]	0.84 [0.16 to 0.96]	0.77 [0.01 to 0.94]	0.75 [0.00 to 0.94]	0.77 [0.07 to 0.94]	0.81 [0.13 to 0.95]
BS/DS time ratio	0.47 [− 0.65 to 0.86]	0.92 [0.66 to 0.98]	0.85 [0.42 to 0.96]	0.76 [0.12 to 0.94]	0.64 [− 0.27 to 0.91]	0.59 [− 0.46 to 0.89]	0.74 [0.05 to 0.93]
DS/FT time ratio	0.55 [− 1.01 to 0.89]	0.72 [− 0.21 to 0.93]	− 0.53 [− 10.78 to 0.68]	0.08 [− 2.22 to 0.76]	0.02 [− 1.57 to 0.72]	0.46 [− 1.62 to 0.87]	0.92 [0.69 to 0.98]
Backswing peak (m s^−2^)	0.91 [0.64 to 0.98]	0.98 [0.92 to 1.00]	0.96 [0.79 to 0.99]	0.97 [0.87 to 0.99]	0.96 [0.78 to 0.99]	0.97 [0.82 to 0.99]	0.96 [0.74 to 0.99]
Downswing peak (m s^−2^)	0.72 [− 0.14 to 0.93]	0.88 [0.51 to 0.97]	0.92 [0.68 to 0.98]	0.95 [0.81 to 0.99]	0.98 [0.90 to 0.99]	0.98 [0.91 to 0.99]	0.97 [0.88 to 0.99]
Backswing peak time (s)	0.51 [− 1.34 to 0.88]	0.49 [− 0.51 to 0.86]	0.57 [− 0.39 to 0.89]	0.48 [− 0.60 to 0.86]	0.48 [− 0.62 to 0.86]	0.44 [− 0.73 to 0.85]	0.55 [− 0.45 to 0.88]
Downswing peak time (s)	0.91 [0.64 to 0.98]	0.85 [0.46 to 0.96]	0.91 [0.65 to 0.98]	0.89 [0.57 to 0.97]	0.90 [0.62 to 0.97]	0.91 [0.66 to 0.98]	0.91 [0.65 to 0.98]
Total resultant velocity (m s^−1^)	− 1.33 [− 244.7 to 0.62]	0.27 [− 1.10 to 0.80]	0.32 [− 0.64 to 0.80]	0.32 [− 0.66 to 0.80]	0.29 [− 0.62 to 0.78]	0.29 [− 0.64 to 0.79]	0.19 [− 0.75 to 0.75]
Backswing resultant velocity (m s^−1^)	0.51 [− 1.10 to 0.88]	0.68 [− 0.30 to 0.92]	0.69 [− 0.14 to 0.92]	0.73 [− 0.11 to 0.93]	0.62 [− 0.33 to 0.90]	0.63 [− 0.31 to 0.91]	0.63 [− 0.32 to 0.91]
Downswing resultant velocity (m s^−1^)	− 0.05 [− 1.73 to 0.70]	0.39 [− 0.72 to 0.83]	0.45 [− 0.71 to 0.85]	0.37 [− 0.88 to 0.83]	0.28 [− 1.07 to 0.80]	0.19 [− 1.34 to 0.78]	0.22 [− 1.31 to 0.79]

The 95% confidence intervals are reported in brackets.

BS: backswing; DS: downswing; FT: follow-through.

**Table 3:** The standard error of the measurement for accelerometer-related variables.

	Number of trials						
	1	5	10	15	20	25	30
Putt time (s)	0.26	0.18	0.19	0.22	0.22	0.21	0.20
Backswing time (s)	0.10	0.05	0.09	0.11	0.13	0.14	0.11
Downswing time(s)	0.09	0.06	0.05	0.06	0.06	0.06	0.05
Follow-through time (s)	0.34	0.13	0.11	0.11	0.10	0.09	0.10
Swing time (s)	0.10	0.09	0.12	0.15	0.16	0.15	0.13
BS/DS time ratio	0.14	0.09	0.14	0.17	0.23	0.24	0.18
DS/FT time ratio	0.30	0.11	0.36	0.23	0.19	0.29	0.12
Backswing peak (m s^−2^)	0.38	0.18	0.23	0.21	0.25	0.22	0.25
Downswing peak (m s^−2^)	0.85	0.45	0.34	0.27	0.18	0.18	0.20
Backswing peak time (s)	0.05	0.08	0.11	0.12	0.13	0.14	0.11
Downswing peak time (s)	0.07	0.07	0.05	0.05	0.05	0.05	0.05
Total resultant velocity (m s^−1^)	70.69	28.07	25.71	26.24	27.38	26.71	28.79
Backswing resultant velocity (m s^−1^)	8.93	5.05	4.33	4.02	5.85	5.43	5.09
Downswing resultant velocity (m s^−1^)	23.09	12.75	12.37	13.42	14.01	14.76	14.27

BS: backswing; DS: downswing; FT: follow-through.

**Table 4:** Minimum detectable changes in accelerometer-related variables.

	Number of trials						
	1	5	10	15	20	25	30
Putt time (s)	0.73	0.50	0.53	0.60	0.61	0.58	0.57
Backswing time (s)	0.29	0.15	0.25	0.31	0.36	0.38	0.30
Downswing time(s)	0.24	0.17	0.14	0.16	0.16	0.15	0.15
Follow-through time (s)	0.93	0.37	0.31	0.31	0.29	0.26	0.27
Swing time (s)	0.28	0.25	0.33	0.40	0.43	0.42	0.37
BS/DS time ratio	0.40	0.25	0.38	0.47	0.63	0.66	0.49
DS/FT time ratio	0.82	0.30	1.00	0.63	0.54	0.79	0.34
Backswing peak (m s^−2^)	1.05	0.49	0.63	0.59	0.69	0.62	0.69
Downswing peak (m s^−2^)	2.36	1.25	0.94	0.74	0.50	0.50	0.56
Backswing peak time (s)	0.13	0.23	0.30	0.34	0.36	0.38	0.30
Downswing peak time (s)	0.20	0.18	0.15	0.15	0.14	0.13	0.14
Total resultant velocity (m s^−1^)	195.94	77.79	71.27	72.74	75.89	74.04	79.79
Backswing resultant velocity (m s^−1^)	24.76	14.01	12.00	11.15	16.22	15.06	14.12
Downswing resultant velocity (m s^−1^)	63.99	35.35	34.29	37.20	38.82	40.91	39.54

BS: backswing; DS: downswing; FT: follow-through.

Finally, in Table 5, the mean and standard deviation of the test and retest results for each variable are shown. It should be noted that the trials used to compute these variables were, following the results of Table 2, 18 trials and 28 trials for score (0–10 and 0–3, respectively) as well as 7 trials for accelerometer-related variables.

**Table 5:** Reliability values of the accelerometer parameters using the required number of trials for the computation.

Variable	Test	Retest	t_9_	Adjusted p-value	ICC	SEM	MDC
Score (0–10)	5.64 (0.72)	6.13 (1.18)	− 1.65	0.97	0.67	0.55	1.52
Score (0–3)	2.06 (0.37)	1.91 (0.28)	1.88	0.91	0.80	0.15	0.41
Putt time (s)	1.46 (0.34)	1.69 (0.32)	− 2.42	0.62	0.67	0.19	0.53
Backswing time (s)	0.4 (0.15)	0.5(0.23)	− 2.40	0.62	0.81	0.08	0.23
Downswing time(s)	0.52 (0.13)	0.57 (0.13)	− 1.71	0.97	0.86	0.05	0.14
Follow-through time (s)	0.52 (0.11)	0.6 (0.09)	− 1.57	0.97	− 0.27	0.12	0.32
Swing time (s)	0.92 (0.26)	1.07 (0.3)	− 2.65	0.44	0.83	0.11	0.32
BS/DS time ratio	0.81 (0.24)	0.93 (0.38)	− 1.98	0.87	0.87	0.11	0.32
DS/FT time ratio	1.08 (0.17)	0.99 (0.23)	1.28	0.97	0.65	0.12	0.33
Backswing peak (m s^−2^)	2.51 (1.07)	2.8 (1.38)	− 2.19	0.74	0.96	0.25	0.70
Donwswing peak (m s^−2^)	5.91 (1.33)	5.96 (1.16)	− 0.21	1	0.94	0.31	0.86
Backswing peak time (s)	0.18 (0.05)	0.3 (0.22)	− 1.89	0.91	0.25	0.12	0.33
Donwswing peak time (s)	0.45 (0.01)	0.48 (0.19)	− 1.01	1	0.91	0.05	0.14
Total resultant velocity (m s^−1^)	234.67 (46.98)	264.30 (15.18)	− 2.06	0.84	0.21	27.65	76.65
Backswing resultant velocity (m s^−1^)	30.53 (8.34)	35.86 (8.14)	− 2.34	0.62	0.69	4.57	12.68
Downswing resultant velocity (m s^−1^)	107.19 (21.14)	116.95 (12.6)	− 1.47	0.97	0.40	13.04	36.13

The test and retest data are expressed as the mean (standard deviation).

ICC: intraclass correlation coefficient; SEM: standard error of the measurement; MDC: minimum detectable change; BS: backswing; DS: downswing; FT: follow-through.

## Discussion

In this manuscript, a test–retest analysis to determine the number of trials required to assess performance and biomechanical variables during putting has been performed for the first time. It should be noted that the participants used in this study were medium-to-high handicap golfers; therefore, the applicability of these results is restricted to this wide population. As the level of players increases, it is possible to reach good reliability indices with fewer trials, and vice versa, due to the greater variability of novice athletes^24^. This has been pointed out in studies examining the reliability of full-swing performance variables in low-handicap^16^ and medium-to-high handicap players^26^.

The results of the present study have important implications for researchers focused on analyzing learning methods to improve the putting performance of novice players. To date, several motor learning experiments have used putt as the skill to improve and quantify the performance of participants using a procedure similar to that presented in the current study^7–14^. Nevertheless, the number of trials used to quantify performance was arbitrarily selected, and we cannot be confident in some of these manuscripts that the variables provided were reliably measured. With the data provided in the present study, future researchers can establish the number of putts (i.e., 28) and the procedure to measure performance (dartboard with 3 levels) while ensuring that the variables are reliably measured. Furthermore, this study provides a methodological framework for trainers who want to correctly measure the performance of players as a tool to determine its evolution. Assessing performance in golf has traditionally been performed by counting the number of strokes required to complete a round. However, it is widely acknowledged that there is significant variability in performance during a round^27^, particularly among amateur players. Therefore, the current method of assessing players' handicaps relies on scores of up to 20 completed rounds. Moreover, while the handicap is a valuable metric for evaluating players' overall performance, it may be advantageous to develop systems that assess specific aspects of the game, such as driving, approach shots, and putting. Some systems can quantify crucial parameters related to driver and approach shots, and reliability studies have determined the number of strokes required to obtain reproducible measurements^28^. Regrettably, no such studies have been conducted in putting skills to date. Therefore, with the measurement protocol proposed in this study, practitioners can measure the performance of amateur players specifically during putting performance and carry out a follow-up of their improvements due to class sessions.

Putting performance was measured in the present study using a dartboard with 10 concentric circles, so the score of each shot was between 0 and 10 points. Nevertheless, as explained in the methods section, the score was also coded as 0–3 points. Using these scores, it was found that the required number of trials to reach reliable data were 18 and 28 for the 0–10-point and 0–3-point dartboard respectively (after the subjects became familiarized with the measurement protocol). It should be considered that the reliability values for 28 trials and the 0–3 system score were greater (ICC = 0.8, SEM = 0.15 and MDC = 0.41) than those obtained for 18 trials and the 0–10 score range (ICC = 0.67, SEM = 0.55, and MDC = 1.52). Then, the overall recommendation that can be made based on these results is to use the 0–3 score system and 28 putts. It is important to consider that the concentric circles in the 0–3 system had a diameter of 24 cm, and this diameter should be used to design the dartboard to measure putting performance to follow the recommendations of the present study. These results make sense for several reasons. First, medium-to-high handicap players present high variability in the performance of each putt stroke if they are considered independently; therefore, it is necessary to perform a set of 28 putts to measure performance reliably. Second, as the diameter of each circle (score) increases, the difference in the putt performance (in distance to the hole) increases to obtain a different score. Thus, the greater the diameter of the circle is, the more reliable the measure of putt performance. One might consider increasing the diameter of the circles even further to obtain more reliable scores more quickly (with fewer trials). It should be noted, however, that as the circle size increases, the sensitivity of the score decreases, and undesirable effects may occur, such as failure to detect significant changes that are important from a practical point of view.

Schweizer et al. assessed the radial error as a performance variable in a similar experimental procedure and task to those described in our study. Specifically, the participants were young adults without specific experience in golf who performed 14 trials from a distance of 2 m. They performed a reliability analysis of just one experimental session using the Spearman-Brown procedure and found that 14 putts were, in their own words, not enough to provide reliable results (ICC = 0.71). They also estimated that 22 putts are needed to reach an ICC of 0.8 for the radial distance variable. This number is slightly lower than that reported in the present study using a test–retest approach, and it should be considered that the scale of measurement of Schweizer et al. is continuous and therefore more sensitive to changes than our 0–3 score scale^17^. Nevertheless, test–retest studies could increase the variability of putts since subjects’ conditions can differ from one day to another due to several factors (e.g., sleep quality)^29,30^. Thus, in our opinion, this protocol can be more appropriate for performing reliability recommendations for putting performance measurements than just reliability analysis within a unique experimental session.

A review of the published manuscripts in which putting performance was measured revealed high variability in the protocols used. Most of the manuscripts used putts of 2–3 m^8,9,14,31^, but some of them used longer distances (i.e., up to 9 m)^32,33^. Moreover, how performance is measured ranges from distance to the hole (a more sensible measure)^8,14,32^ to the percentage of holed putts (a less sensible measure)^31,34^. Considering only the manuscripts using putts of approximately 2 m, some of them performed more than 28 putts^10,35,36^, but others performed less than our recommendation for medium-to-high handicap players^31,37–40^. Thus, our results provide a reliable measurement protocol for putting performance measurements to guide future studies to reach reliable results.

Regarding accelerometer variables, it was found that 7 trials were enough to assess several of them with good-to-excellent reliability (i.e., ICC = 0.65–0.96). Nevertheless, it should be noted that other variables did not reach the ICC, SEM or MDC values to ensure that the measurements were reliable. These variables were follow-through time, backswing peak time, total resultant velocity, and downswing resultant velocity. As only one of the area under the curve variables reaches acceptable values (i.e., backswing resultant velocity), it can be considered that the energy under the curve of the resultant acceleration during putting is not reliable in medium-to-high handicap players. Instead, peak acceleration has been established as a reliable parameter for snatch lift performance^41^, running impact force monitoring^42^ and jumping or landing impact forces^43^. In our case, the peak acceleration, both in the backswing and downswing directions, demonstrated excellent reliability. It makes sense that peak variables are more reliable than others, such as velocity variables calculated from accelerations for medium to high handicap players, as the peaks can be determined by the subject's maximum capability to generate accelerations. However, the area under the curve variables are determined by both the duration of the putt phase and the resultant vector accelerations. Therefore, the subject can accelerate and decelerate more in a given phase of the putt, thus increasing the area under the curve variable but achieving the same velocity at impact. This fact has been found previously in accelerometer-derived variables acquired during a countermovement jump^44^. It was found that the peak force is a more reliable variable than the velocity and power. Therefore, it is recommended that peak variables are evaluated rather than variables related to the area under the resultant acceleration curve. Therefore, to provide a recommendation based on the evidence of this study, to evaluate the biomechanics using accelerometers in medium-to-high handicap players, the put time, swing time, backswing, and downswing time, as well as the peak acceleration in backswing and downswing, should be computed.

This manuscript has some limitations that should be underlined. First, there are accelerometer-related variables that have not reached reliable values, and it is possible that by increasing the number of putts above 30, good reliability indices could be reached for these variables. Moreover, this study determined the performance reliability using a dartboard score, but in the literature, other methodologies, such as the radial distance or percentage of holed putts, were not assessed. Additionally, distance and the handicap of the player can be important factors for determining reliability. We used a distance of 2 m, and the participants were medium-to-high handicap players. All these variables can influence the reliability scores; therefore, our results should be extrapolated only to this population and under the experimental conditions described.

It can be concluded that putting performance in medium-to-high handicap golf players can be reliably measured using a dartboard painted in the putting mat with concentric circles 24 cm in diameter, providing a putting score from 0 to 3 points, a 2-m distance from the start point to the center of the target and performing 28 attempts. Most accelerometer-derived variables can be reliably measured only by performing 7 trials, but some parameters do not reach reliable values. This confirms partially our hypothesis, as both performance and several of the biomechanical variables can be reliably measured using the protocols recommended in this manuscript.

## Methods

### Participants

The sample size was determined using the equation proposed by Bonett^45^, and the ICC value (i.e., 0.8) obtained by Schweizer et al. if participants performed 22 putts to compute the radial distance to the hole^17^. The significance level was set at p = 0.05 with 80% statistical power. Accordingly, with this analysis, ten subjects participated in the current study. A nonprobabilistic sampling method was used to obtain the study sample, which was composed of 10 golf players (2 women) with a mean handicap of 31.23 (6.63). The players were 55.67 (13.64) years old, 78.4 (11.4) kg in weight, 1.75 (7.95) m in height and 25.62 (2.44) kg/m^2^ in body mass index. To carry out the convenience sampling, the following inclusion criteria were used: (i) handicap players had 18 to 36 strokes, (ii) in the last six months before the study, golf players had not suffered any injury, and (iii) participants could not have previously suffered from a disease that influenced the heart rate.

The subjects provided informed consent before participating in the study. The protocols used in this research received ethical approval from the local ethics committee (PI:033/2022). These protocols also met the requirements set out in the Declaration of Helsinki, 1975, which was subsequently reviewed in 2008.

### Tasks and apparatus

Two within-subject experimental sessions were carried out to determine the test–retest reliability. Both sessions had the same design, first, 10 min of warm-up guided by a professional graduate in physical activity and sport and, second, the experimental task was realized. The warm-up consisted of the joint mobility of the ankles (clockwise and counterclockwise circles), knees (flexion–extension), hips (clockwise and counterclockwise circles), shoulders (rotating forward and backward), elbows (flexion–extension) and wrists (clockwise and counterclockwise circles) (30 s each), as well as ten putt swing trials without hitting the ball. Finally, the subjects performed ten putt swings hitting the ball under the same conditions as the assessed task (see below), which served as the specific warm-up and familiarization with the task.

The experimental task consisted of performing 30 shots to stop the golf ball at the center of a dartboard painted on a golf putting mat surface. The mat surface was 1 m wide and 5 m long, and the distance between the start point and the center of the dartboard was 2 m. The dartboard has 10 concentric circles, each 8 cm in diameter larger than the previous one. Thus, the size of the dartboard was 80 × 80 cm. The subjects performed three rounds of 10 putts, and a rest between blocks (1–2 min) was allowed to avoid fatigue.

An Xsens Dot (Motion Technologies, Enschede, Netherlands) was placed in the head of the putter of the participants to acquire acceleration signals during golf putt swings. This inertial measurement system has reduced dimensions (i.e., 63 × 30 × 22 mm) and was secured to the putter using adhesive tape. The free acceleration signals were acquired using the Xsens DOT mobile APP with a 60 Hz sample frequency.

**Figure 2:**
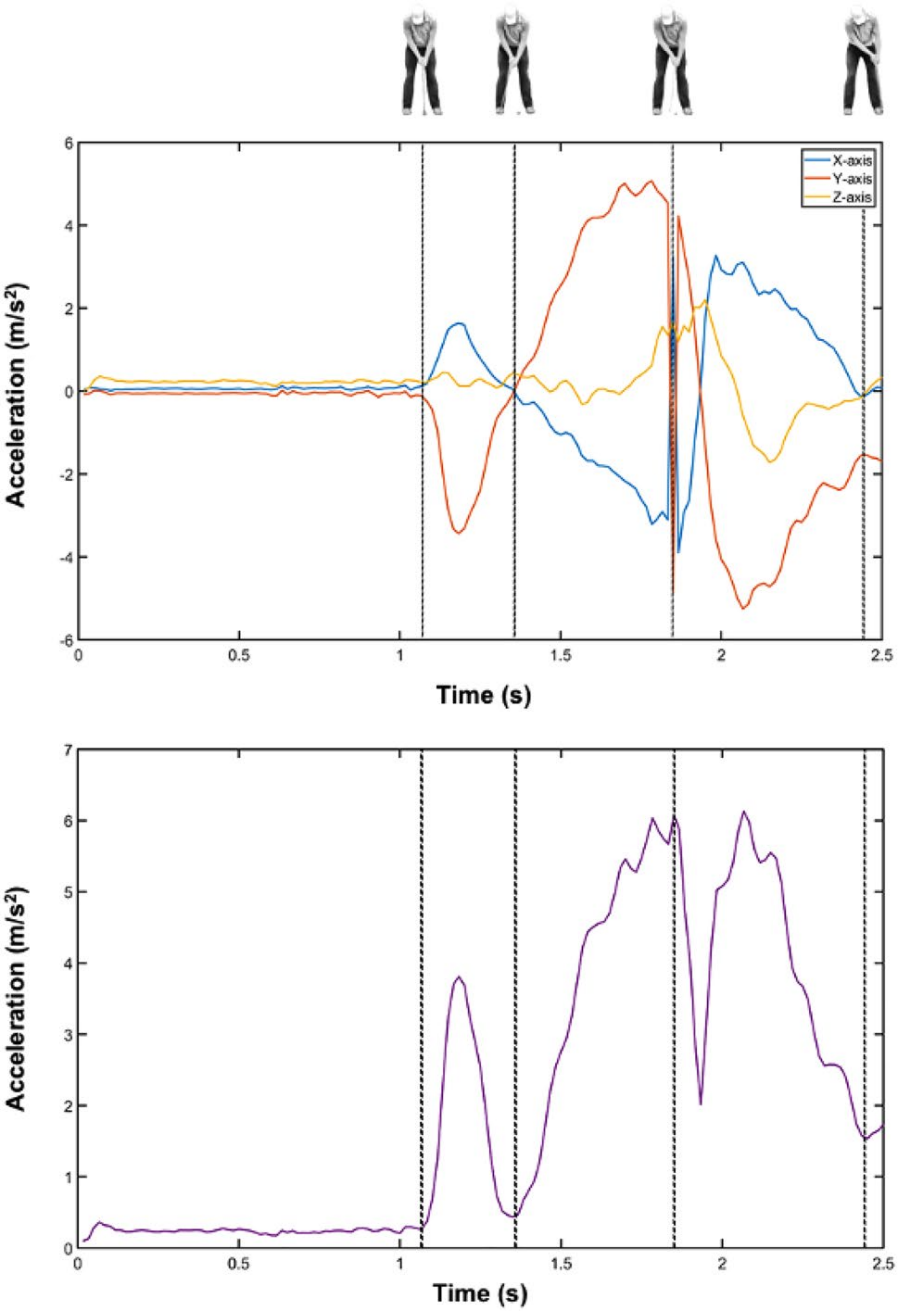
Example of putting acceleration signals on the three axes (upper layer) and the resultant acceleration (lower layer).

When the subjects were placed in the initial position (putt stance), the researcher started the acceleration measurement through the Xsens app, and once the acceleration signals started to be acquired, the researcher gave verbal instructions to the participant to start the trial. The participants were instructed to maintain the finish position at the end of the swing, and the recording was stopped when the participants reached this position. Figure 2 shows an example of the free accelerations on the three axes as well as the resultant vector.

### Data processing

The data processing of acceleration signals was performed using MATLAB R2021b (MathWorks, Natick, MA, USA). First, the resultant acceleration was computed as the root mean square of the sum of the squares of the acceleration in each axis. Then, signals were segmented using a manual and automatic combined process. The beginning and the end of the putt swings were determined manually, while the top of the backswing and the impact point were determined automatically. The latter was determined as the maximum value of the resultant vector signal filtered with a Butterworth high-pass filter (2nd order; 15 Hz cutoff frequency). Last, the top of the backswing was established as the lowest resultant acceleration between the impact point and the highest acceleration during the first 300 ms of the backswing. Once the points were established, a visual inspection was performed, and if a point was incorrectly detected, it was manually established. Figure 2 shows an example of putt acceleration signals and the points corresponding to the beginning of the backswing, the top of the backswing, the impact point and the end of the swing (dashed vertical lines).

At this point, the resultant vector was low pass filtered with a Butterworth filter and a cutoff frequency of 10 Hz. Then, some of the acceleration parameters computed by Jensen et al.^23^ were extracted. A description of these variables is provided in Table 6.

**Table 6:** Description of accelerometer-related variables during putting.

Variable	Units	Description	Equation
Putt time	(s)	Time elapsed from the beginning of the backswing until the finish of the swing	$\left({t}_{4}-{t}_{1}\right)$
Backswing time	(s)	Time elapsed between the beginning until the top of the backswing	$\left({t}_{2}-{t}_{1}\right)$
Downswing time	(s)	Time elapsed between the top of the backswing and the impact point	$\left({t}_{3}-{t}_{2}\right)$
Follow-through time	(s)	Time elapsed between the impact points and the finish of the swing	$\left({t}_{4}-{t}_{3}\right)$
Swing time	(s)	Time elapsed between the start of the backswing and the impact point	$\left({t}_{3}-{t}_{1}\right)$
BS/DS time ratio	–	Ratio of time between backswing and downswing	$\frac{\left({t}_{2}-{t}_{1}\right)}{\left({t}_{3}-{t}_{2}\right)}$
DS/FT time ratio	–	Ratio of time between downswing and follow-through	$\frac{\left({t}_{3}-{t}_{2}\right)}{\left({t}_{4}-{t}_{3}\right)}$
Backswing peak	(m s^−2^)	Maximal acceleration during the backswing	$\mathop {\max }\limits_{{t1 < t < t2}} ACC\left\{t\right\}$
Downswing peak	(m s^−2^)	Maximal acceleration during the downswing	$\mathop {\max }\limits_{{t2<t<t3}} ACC\left\{t\right\}$
Backswing peak time	(s)	Time on which the maximal acceleration of the backswing occurred	$t\subset \left[{t}_{1},{t}_{2}\right]:{ACC}_{t}=\mathop {\max }\limits_{{t1<t<t2}} ACC\left\{t\right\}$
Downswing peak time	(s)	Time on which the maximal acceleration of the downswing occurred	$t\subset \left[{t}_{2},{t}_{3}\right]:{ACC}_{t}=\mathop {\max }\limits_{{t2<t<t3}}ACC\left\{t\right\}$
Total resultant velocity	(m s^−1^)	Area under the curve of the whole acceleration signal	$\sum_{t=0}^{t4}{ACC}_{t}$
Backswing resultant velocity	(m s^−1^)	Area under the curve of the acceleration signal during the backswing	$\sum_{t=0}^{t2}{ACC}_{t}$
Downswing resultant velocity	(m s^−1^)	Area under the curve of the acceleration signal during the downswing	$\sum_{t=t2}^{t3}{ACC}_{t}$

t_1_: instant of time at which the swing starts; t_2_: instant of time at which the top of the backswing is reached; t_3_: instant of time when the impact with the ball occurs; t_4_: instant of time at which the swing ends (each instant time is computed as the data point at which the event occurs divided by the sampling frequency); ACC: acceleration (resultant vector).

### Statistical analysis

The software used to perform the statistical analysis was MATLAB R2021b (MathWorks, Natick, MA, USA). In this study, the following test–retest parameters were computed: (i) the intraclass correlation coefficient [*A,k* model^46^] to obtain relative reliability by examining the relationship between repeated measurements; (ii) the standard error of the measurement (SEM) as an absolute reliability value; and (iii) the minimum detectable change (MDC), defined as the minimum magnitude of change that is required to know a true change in improvement due to variability in performance or measurement error.

Every parameter mentioned previously was computed considering only the first swing on both experimental sessions as well as the mean value from 2 to 30 swings. Other studies have carried out this process to clarify the amount of swings required to achieve acceptable reliability of full swing-related variables^15^. An ICC greater than 0.9 indicated excellent reliability, values between 0.75 and 0.9 demonstrated good reliability, values between 0.5 and 0.75 indicated moderate reliability, and values lower than 0.5 indicated poor reliability^47^.

To determine the number of trials required to compute each performance variable, the Required Trials Index (RTI; Eq. 1) was used. This index allows us to determine the best number of trials by taking into consideration the relationship between the number of trials and the reliability value.

1 $${RTI}_{i}= \frac{\left(\frac{\left({ICC}_{1}-{ICC}_{i}\right)}{{ICC}_{1}}-1\right)\cdot 100}{(\sqrt{i}+1)}$$

*ICC* is the intraclass correlation coefficient, *i* is the number of swings utilized to calculate the ICC, and *ICC*_*1*_ is the ICC obtained from the first trial. It should be noted that the RTIi values are below 0, and the lower the RTIi is, the greater the relationship between reliability and the number of trials needed. This equation was also used to calculate the same index for SEM and MDC values, but for these two parameters, the -1 observed in the numerator was modified to + 1. In these cases, an RTI < 50 indicates that the SEM or MDC using more than one trial results in lower reliability than using just one trial, while an RTI > 50 indicates that the reliability using *i* trials is better than that using only one trial. Therefore, the number of swings with the lowest RTI in the ICC and highest RTI in the SEM and MDC were chosen for every performance parameter as the best ratio between reliability and the number of trials.

Moreover, since only ten subjects participated in this study, we calculated and provided the coefficient of variation of each subject for each of the variables considered in this study as supplementary material. This parameter was calculated considering the experimental protocol from trials 1 to 30, as described previously. These data can be of interest to readers since they can provide an overview of the effect of increasing the number of trials to measure performance and biomechanical variables on an individual basis.

The mean values and standard deviation of every performance variable were calculated using the required number of swings that, according to the RTI, were 7 for acceleration-related variables, 18 for the score obtained on a 0–10 dartboard and 28 for the score in the 0–3 dartboard range. Finally, Student’s t-test for related samples was used to compare the mean of the test and the retest for each variable. The level of significance was set at p = 0.05.

### Supplementary Information

Supplementary Information.

## Data Availability

The datasets generated and/or analyzed during the current study are available from the corresponding author upon reasonable request.
