# Supplementary material for: Test–retest reliability of putting-related variables in medium-to-high handicap golf players
Source: Sci Rep. 2024 May 20;14:11516. doi: 10.1038/s41598-024-62183-z (PMC11106066; doi:10.1038/s41598-024-62183-z)
Supplement: Supplementary file 1 — Supplementary Information. [file 41598_2024_62183_MOESM1_ESM.pdf]

Supplementary information

In the following tables “Subj” refers to subject (e.g., Subj 1 relates to Subject 1). The trial number is also specified (e.g., 1 trial).

CV score 0-10

	**1 trial**	**2 trial**	**3 trial**	**4 trial**	**5 trial**	**6 trial**	**7 trial**	**8 trial**	**9 trial**	**10 trial**	**11 trial**	**12 trial**	**13 trial**	**14 trial**	**15 trial**	**16 trial**	**17 trial**	**18 trial**	**19 trial**	**20 trial**	**21 trial**	**22 trial**	**23 trial**	**24 trial**	**25 trial**	**26 trial**	**27 trial**	**28 trial**	**29 trial**	**30 trial**
**Subj 1**	0.18	0.08	0.06	0.10	0.04	0.07	0.21	0.22	0.25	0.30	0.26	0.28	0.22	0.21	0.19	0.18	0.16	0.17	0.18	0.21	0.22	0.21	0.18	0.16	0.16	0.16	0.12	0.10	0.09	0.11
**Subj 2**	1.06	0.39	0.39	0.42	0.33	0.43	0.34	0.27	0.12	0.07	0.04	0.05	0.07	0.08	0.09	0.11	0.11	0.09	0.07	0.08	0.08	0.09	0.08	0.07	0.07	0.09	0.12	0.13	0.16	0.18
**Sub3**	0.18	0.40	0.56	0.45	0.52	0.51	0.40	0.38	0.35	0.31	0.28	0.27	0.26	0.22	0.22	0.21	0.19	0.19	0.20	0.21	0.21	0.19	0.20	0.19	0.18	0.17	0.19	0.19	0.20	0.19
**Subj 4**	0.00	0.31	0.44	0.37	0.35	0.35	0.38	0.36	0.33	0.35	0.31	0.25	0.23	0.23	0.22	0.19	0.18	0.15	0.16	0.20	0.15	0.14	0.12	0.13	0.11	0.10	0.13	0.13	0.13	0.13
**Subj 5**	0.57	0.33	0.17	0.30	0.04	0.09	0.11	0.14	0.06	0.18	0.16	0.13	0.15	0.20	0.17	0.16	0.11	0.10	0.08	0.09	0.09	0.09	0.09	0.11	0.14	0.11	0.16	0.13	0.13	0.12
**Subj 6**	0.24	0.06	0.04	0.10	0.00	0.05	0.09	0.10	0.01	0.10	0.08	0.03	0.01	0.03	0.03	0.04	0.03	0.00	0.00	0.02	0.05	0.02	0.05	0.04	0.01	0.03	0.04	0.03	0.03	0.05
**Subj 7**	0.00	0.64	0.00	0.20	0.11	0.03	0.05	0.02	0.02	0.01	0.04	0.08	0.10	0.03	0.05	0.07	0.08	0.12	0.14	0.14	0.18	0.18	0.19	0.18	0.20	0.17	0.14	0.15	0.15	0.16
**Subj 8**	1.41	0.62	0.47	0.40	0.18	0.16	0.09	0.03	0.01	0.03	0.04	0.05	0.03	0.00	0.01	0.03	0.03	0.04	0.04	0.03	0.04	0.06	0.09	0.09	0.08	0.08	0.07	0.07	0.08	0.09
**Subj 9**	0.11	0.10	0.04	0.11	0.12	0.08	0.08	0.03	0.04	0.01	0.07	0.01	0.03	0.07	0.08	0.07	0.06	0.06	0.05	0.05	0.05	0.03	0.02	0.03	0.04	0.02	0.03	0.01	0.00	0.02
**Subj 10**	0.28	0.25	0.09	0.06	0.07	0.11	0.08	0.05	0.08	0.11	0.12	0.13	0.18	0.16	0.11	0.09	0.09	0.10	0.08	0.11	0.11	0.09	0.08	0.08	0.08	0.07	0.06	0.07	0.07	0.08

CV score 0-3

	1 trial	2 trials	3 trials	4 trials	5 trials	6 trials	7 trials	8 trials	9 trials	10 trials	11 trials	12 trials	13 trials	14 trials	15 trials	16 trials	17 trials	18 trials	19 trials	20 trials	21 trials	22 trials	23 trials	24 trials	25 trials	26 trials	27 trials	28 trials	29 trials	30 trials
**Subj 1**	0.28	0.13	0.09	0.14	0.13	0.16	0.28	0.28	0.33	0.36	0.29	0.31	0.26	0.24	0.22	0.21	0.17	0.18	0.19	0.21	0.22	0.20	0.17	0.15	0.15	0.15	0.11	0.09	0.09	0.11
**Subj 2**	1.41	0.47	0.39	0.47	0.35	0.42	0.34	0.24	0.11	0.05	0.08	0.08	0.07	0.09	0.09	0.11	0.10	0.07	0.05	0.07	0.06	0.08	0.07	0.07	0.07	0.08	0.11	0.13	0.16	0.18
**Subj 3**	0.28	0.47	0.64	0.53	0.57	0.51	0.41	0.39	0.35	0.32	0.29	0.27	0.24	0.22	0.23	0.20	0.19	0.17	0.18	0.18	0.17	0.17	0.17	0.16	0.15	0.14	0.15	0.15	0.16	0.16
**Subj 4**	0.00	0.35	0.47	0.35	0.37	0.39	0.40	0.34	0.33	0.36	0.30	0.26	0.24	0.22	0.20	0.16	0.15	0.12	0.14	0.17	0.11	0.11	0.08	0.09	0.09	0.09	0.11	0.11	0.11	0.11
**Subj 5**	0.47	0.00	0.00	0.13	0.11	0.09	0.08	0.00	0.06	0.05	0.05	0.04	0.07	0.14	0.10	0.09	0.03	0.03	0.00	0.02	0.02	0.02	0.02	0.04	0.07	0.04	0.09	0.05	0.05	0.05
**Subj 6**	0.00	0.00	0.11	0.16	0.00	0.05	0.09	0.12	0.00	0.07	0.07	0.03	0.00	0.03	0.03	0.05	0.02	0.00	0.00	0.02	0.05	0.02	0.05	0.03	0.00	0.03	0.03	0.03	0.02	0.04
**Subj 7**	0.00	0.71	0.00	0.28	0.11	0.00	0.00	0.06	0.00	0.04	0.04	0.10	0.12	0.06	0.08	0.10	0.09	0.14	0.15	0.14	0.19	0.18	0.19	0.18	0.19	0.17	0.15	0.16	0.17	0.17
**Subj 8**	1.41	0.71	0.47	0.40	0.18	0.14	0.11	0.05	0.00	0.03	0.06	0.08	0.07	0.05	0.02	0.00	0.00	0.02	0.02	0.02	0.03	0.06	0.08	0.08	0.07	0.08	0.07	0.07	0.07	0.08
**Subj 9**	0.00	0.16	0.00	0.18	0.07	0.00	0.05	0.00	0.00	0.04	0.04	0.03	0.03	0.09	0.08	0.07	0.04	0.04	0.04	0.03	0.03	0.03	0.01	0.03	0.04	0.01	0.02	0.01	0.01	0.02
**Subj 10**	0.47	0.28	0.16	0.09	0.14	0.17	0.15	0.12	0.14	0.16	0.18	0.19	0.22	0.20	0.15	0.15	0.14	0.15	0.12	0.15	0.14	0.12	0.10	0.10	0.09	0.07	0.06	0.07	0.08	0.08

CV putt time (measured in s)

	1 trial	2 trials	3 trials	4 trials	5 trials	6 trials	7 trials	8 trials	9 trials	10 trials	11 trials	12 trials	13 trials	14 trials	15 trials	16 trials	17 trials	18 trials	19 trials	20 trials	21 trials	22 trials	23 trials	24 trials	25 trials	26 trials	27 trials	28 trials	29 trials	30 trials
**Subj 1**	0.19	0.12	0.02	0.04	0.02	0.06	0.09	0.07	0.09	0.09	0.07	0.08	0.07	0.07	0.06	0.05	0.04	0.06	0.07	0.08	0.08	0.08	0.09	0.09	0.09	0.09	0.09	0.10	0.09	0.10
**Subj 2**	0.04	0.09	0.05	0.12	0.13	0.10	0.10	0.08	0.07	0.07	0.07	0.08	0.09	0.08	0.09	0.09	0.09	0.08	0.08	0.07	0.07	0.07	0.07	0.07	0.07	0.08	0.08	0.08	0.08	0.08
**Subj 3**	0.16	0.11	0.10	0.09	0.07	0.06	0.04	0.05	0.06	0.05	0.07	0.07	0.05	0.05	0.05	0.05	0.05	0.05	0.05	0.05	0.05	0.05	0.05	0.05	0.04	0.03	0.04	0.03	0.04	0.04
**Subj 4**	0.36	0.35	0.37	0.33	0.34	0.34	0.35	0.36	0.36	0.36	0.36	0.36	0.36	0.37	0.38	0.37	0.38	0.38	0.37	0.37	0.37	0.36	0.37	0.37	0.37	0.37	0.38	0.38	0.39	0.39
**Subj 5**	0.40	0.40	0.41	0.37	0.39	0.41	0.41	0.40	0.40	0.41	0.42	0.42	0.42	0.44	0.45	0.44	0.44	0.42	0.42	0.41	0.42	0.41	0.41	0.39	0.39	0.39	0.40	0.39	0.40	0.40
**Subj 6**	0.05	0.03	0.05	0.05	0.02	0.04	0.04	0.04	0.03	0.03	0.03	0.03	0.04	0.03	0.03	0.03	0.04	0.04	0.04	0.05	0.05	0.05	0.05	0.05	0.05	0.05	0.05	0.05	0.05	0.05
**Subj 7**	0.02	0.01	0.00	0.02	0.00	0.01	0.05	0.08	0.11	0.11	0.11	0.11	0.10	0.10	0.10	0.11	0.10	0.10	0.10	0.10	0.09	0.10	0.09	0.10	0.10	0.10	0.10	0.10	0.10	0.10
**Subj 8**	0.01	0.06	0.04	0.14	0.12	0.15	0.17	0.12	0.15	0.18	0.19	0.20	0.21	0.20	0.23	0.23	0.22	0.23	0.24	0.25	0.25	0.26	0.26	0.25	0.25	0.23	0.23	0.23	0.22	0.21
**Subj 9**	0.05	0.03	0.01	0.01	0.01	0.02	0.01	0.01	0.02	0.04	0.03	0.04	0.04	0.04	0.04	0.03	0.03	0.03	0.03	0.03	0.03	0.02	0.02	0.02	0.01	0.02	0.02	0.02	0.02	0.01
**Subj 10**	0.02	0.00	0.00	0.02	0.04	0.01	0.00	0.01	0.00	0.01	0.01	0.01	0.00	0.00	0.00	0.01	0.02	0.02	0.03	0.03	0.03	0.03	0.03	0.02	0.03	0.03	0.03	0.03	0.04	0.03

CV backswing time (measured in s)

	**1 trial**	**2 trials**	**3 trials**	**4 trials**	**5 trials**	**6 trials**	**7 trials**	**8 trials**	**9 trials**	**10 trials**	**11 trials**	**12 trials**	**13 trials**	**14 trials**	**15 trials**	**16 trials**	**17 trials**	**18 trials**	**19 trials**	**20 trials**	**21 trials**	**22 trials**	**23 trials**	**24 trials**	**25 trials**	**26 trials**	**27 trials**	**28 trials**	**29 trials**	**30 trials**
**Subj 1**	0.00	0.14	0.14	0.13	0.13	0.16	0.18	0.16	0.17	0.16	0.17	0.17	0.16	0.15	0.16	0.16	0.16	0.16	0.15	0.14	0.15	0.15	0.14	0.15	0.14	0.14	0.14	0.15	0.15	0.14
**Subj 2**	0.04	0.05	0.00	0.11	0.09	0.08	0.07	0.04	0.04	0.04	0.05	0.05	0.06	0.07	0.10	0.09	0.09	0.07	0.08	0.08	0.07	0.07	0.07	0.07	0.07	0.08	0.08	0.08	0.08	0.07
**Subj 3**	0.00	0.10	0.04	0.00	0.01	0.02	0.08	0.08	0.08	0.09	0.07	0.06	0.07	0.09	0.08	0.08	0.08	0.08	0.08	0.07	0.08	0.09	0.09	0.09	0.10	0.11	0.11	0.12	0.12	0.12
**Subj 4**	0.45	0.42	0.36	0.36	0.37	0.36	0.37	0.38	0.37	0.36	0.33	0.31	0.29	0.32	0.33	0.32	0.31	0.31	0.32	0.32	0.33	0.32	0.32	0.33	0.33	0.33	0.33	0.33	0.34	0.33
**Subj 5**	0.16	0.22	0.23	0.24	0.29	0.34	0.33	0.31	0.30	0.31	0.29	0.31	0.30	0.29	0.32	0.31	0.31	0.29	0.29	0.28	0.29	0.29	0.28	0.27	0.27	0.27	0.29	0.28	0.28	0.29
**Subj 6**	0.03	0.06	0.05	0.05	0.04	0.03	0.01	0.00	0.00	0.00	0.02	0.01	0.01	0.01	0.01	0.01	0.01	0.02	0.01	0.01	0.01	0.01	0.02	0.02	0.02	0.02	0.02	0.02	0.02	0.02
**Subj 7**	0.06	0.13	0.11	0.10	0.11	0.12	0.13	0.14	0.12	0.11	0.13	0.14	0.12	0.12	0.11	0.10	0.10	0.10	0.11	0.07	0.08	0.08	0.08	0.09	0.08	0.09	0.09	0.09	0.10	0.10
**Subj 8**	0.16	0.05	0.01	0.25	0.05	0.23	0.32	0.23	0.30	0.37	0.39	0.42	0.44	0.44	0.47	0.48	0.48	0.48	0.51	0.53	0.55	0.59	0.57	0.56	0.56	0.51	0.50	0.49	0.45	0.45
**Subj 9**	0.56	0.23	0.25	0.16	0.21	0.09	0.11	0.09	0.15	0.05	0.02	0.01	0.03	0.03	0.02	0.03	0.01	0.01	0.02	0.03	0.03	0.03	0.03	0.07	0.07	0.06	0.07	0.05	0.06	0.05
**Subj 10**	0.17	0.06	0.01	0.01	0.02	0.06	0.15	0.12	0.11	0.07	0.06	0.05	0.05	0.04	0.06	0.07	0.06	0.06	0.14	0.15	0.15	0.15	0.15	0.15	0.14	0.14	0.14	0.17	0.16	0.15

CV downswing time (measured in s)

	**1 trial**	**2 trials**	**3 trials**	**4 trials**	**5 trials**	**6 trials**	**7 trials**	**8 trials**	**9 trials**	**10 trials**	**11 trials**	**12 trials**	**13 trials**	**14 trials**	**15 trials**	**16 trials**	**17 trials**	**18 trials**	**19 trials**	**20 trials**	**21 trials**	**22 trials**	**23 trials**	**24 trials**	**25 trials**	**26 trials**	**27 trials**	**28 trials**	**29 trials**	**30 trials**
**Subj 1**	0.02	0.02	0.01	0.01	0.00	0.00	0.01	0.01	0.00	0.01	0.00	0.01	0.02	0.02	0.01	0.01	0.01	0.01	0.01	0.01	0.01	0.01	0.01	0.01	0.01	0.01	0.01	0.01	0.00	0.00
**Subj 2**	0.07	0.08	0.06	0.05	0.05	0.05	0.06	0.07	0.07	0.07	0.07	0.09	0.09	0.08	0.08	0.08	0.08	0.07	0.07	0.07	0.06	0.06	0.06	0.06	0.07	0.07	0.06	0.07	0.07	0.07
**Subj 3**	0.09	0.02	0.03	0.04	0.05	0.06	0.07	0.07	0.08	0.08	0.08	0.08	0.08	0.07	0.07	0.07	0.07	0.07	0.08	0.08	0.07	0.08	0.07	0.07	0.07	0.07	0.07	0.07	0.07	0.07
**Subj 4**	0.43	0.31	0.33	0.26	0.28	0.31	0.28	0.29	0.30	0.32	0.32	0.35	0.37	0.36	0.36	0.36	0.37	0.37	0.37	0.36	0.35	0.35	0.35	0.34	0.34	0.34	0.34	0.33	0.33	0.34
**Subj 5**	0.31	0.31	0.29	0.29	0.29	0.30	0.33	0.28	0.32	0.32	0.34	0.34	0.35	0.41	0.40	0.40	0.41	0.40	0.41	0.41	0.41	0.41	0.40	0.41	0.41	0.41	0.40	0.40	0.39	0.40
**Subj 6**	0.05	0.02	0.02	0.02	0.01	0.02	0.02	0.02	0.01	0.01	0.02	0.02	0.02	0.02	0.02	0.02	0.02	0.02	0.02	0.02	0.02	0.02	0.02	0.02	0.02	0.02	0.02	0.03	0.03	0.03
**Subj 7**	0.02	0.03	0.01	0.01	0.01	0.01	0.01	0.02	0.02	0.02	0.01	0.01	0.01	0.00	0.00	0.01	0.01	0.01	0.01	0.01	0.01	0.02	0.02	0.02	0.02	0.02	0.02	0.02	0.03	0.03
**Subj 8**	0.02	0.04	0.04	0.06	0.25	0.12	0.09	0.08	0.05	0.04	0.04	0.04	0.05	0.04	0.06	0.05	0.04	0.04	0.03	0.03	0.01	0.04	0.03	0.05	0.04	0.04	0.03	0.00	0.00	0.00
**Subj 9**	0.30	0.20	0.19	0.16	0.17	0.10	0.11	0.10	0.17	0.16	0.13	0.12	0.11	0.11	0.10	0.10	0.09	0.09	0.09	0.08	0.08	0.07	0.07	0.07	0.07	0.07	0.06	0.05	0.06	0.06
**Subj 10**	0.22	0.14	0.15	0.13	0.10	0.07	0.09	0.10	0.09	0.03	0.02	0.03	0.03	0.04	0.04	0.03	0.02	0.02	0.03	0.03	0.03	0.03	0.03	0.03	0.02	0.02	0.03	0.03	0.04	0.04

CV Follow-through time (measured in s)

	**1 trial**	**2 trials**	**3 trials**	**4 trials**	**5 trials**	**6 trials**	**7 trials**	**8 trials**	**9 trials**	**10 trials**	**11 trials**	**12 trials**	**13 trials**	**14 trials**	**15 trials**	**16 trials**	**17 trials**	**18 trials**	**19 trials**	**20 trials**	**21 trials**	**22 trials**	**23 trials**	**24 trials**	**25 trials**	**26 trials**	**27 trials**	**28 trials**	**29 trials**	**30 trials**
**Subj 1**	0.02	0.02	0.01	0.01	0.00	0.00	0.01	0.01	0.00	0.01	0.00	0.01	0.02	0.02	0.01	0.01	0.01	0.01	0.01	0.01	0.01	0.01	0.01	0.01	0.01	0.01	0.01	0.01	0.00	0.00
**Subj 2**	0.07	0.08	0.06	0.05	0.05	0.05	0.06	0.07	0.07	0.07	0.07	0.09	0.09	0.08	0.08	0.08	0.08	0.07	0.07	0.07	0.06	0.06	0.06	0.06	0.07	0.07	0.06	0.07	0.07	0.07
**Subj 3**	0.09	0.02	0.03	0.04	0.05	0.06	0.07	0.07	0.08	0.08	0.08	0.08	0.08	0.07	0.07	0.07	0.07	0.07	0.08	0.08	0.07	0.08	0.07	0.07	0.07	0.07	0.07	0.07	0.07	0.07
**Subj 4**	0.43	0.31	0.33	0.26	0.28	0.31	0.28	0.29	0.30	0.32	0.32	0.35	0.37	0.36	0.36	0.36	0.37	0.37	0.37	0.36	0.35	0.35	0.35	0.34	0.34	0.34	0.34	0.33	0.33	0.34
**Subj 5**	0.31	0.31	0.29	0.29	0.29	0.30	0.33	0.28	0.32	0.32	0.34	0.34	0.35	0.41	0.40	0.40	0.41	0.40	0.41	0.41	0.41	0.41	0.40	0.41	0.41	0.41	0.40	0.40	0.39	0.40
**Subj 6**	0.05	0.02	0.02	0.02	0.01	0.02	0.02	0.02	0.01	0.01	0.02	0.02	0.02	0.02	0.02	0.02	0.02	0.02	0.02	0.02	0.02	0.02	0.02	0.02	0.02	0.02	0.02	0.03	0.03	0.03
**Subj 7**	0.02	0.03	0.01	0.01	0.01	0.01	0.01	0.02	0.02	0.02	0.01	0.01	0.01	0.00	0.00	0.01	0.01	0.01	0.01	0.01	0.01	0.02	0.02	0.02	0.02	0.02	0.02	0.02	0.03	0.03
**Subj 8**	0.02	0.04	0.04	0.06	0.25	0.12	0.09	0.08	0.05	0.04	0.04	0.04	0.05	0.04	0.06	0.05	0.04	0.04	0.03	0.03	0.01	0.04	0.03	0.05	0.04	0.04	0.03	0.00	0.00	0.00
**Subj 9**	0.30	0.20	0.19	0.16	0.17	0.10	0.11	0.10	0.17	0.16	0.13	0.12	0.11	0.11	0.10	0.10	0.09	0.09	0.09	0.08	0.08	0.07	0.07	0.07	0.07	0.07	0.06	0.05	0.06	0.06
**Subj 10**	0.22	0.14	0.15	0.13	0.10	0.07	0.09	0.10	0.09	0.03	0.02	0.03	0.03	0.04	0.04	0.03	0.02	0.02	0.03	0.03	0.03	0.03	0.03	0.03	0.02	0.02	0.03	0.03	0.04	0.04

CV Swing time (measured in s)

	1 trial	2 trials	3 trials	4 trials	5 trials	6 trials	7 trials	8 trials	9 trials	10 trials	11 trials	12 trials	13 trials	14 trials	15 trials	16 trials	17 trials	18 trials	19 trials	20 trials	21 trials	22 trials	23 trials	24 trials	25 trials	26 trials	27 trials	28 trials	29 trials	30 trials
**Subj 1**	0.01	0.06	0.07	0.06	0.07	0.08	0.09	0.08	0.09	0.08	0.09	0.09	0.09	0.09	0.09	0.08	0.07	0.07	0.07	0.07	0.07	0.07	0.07	0.07	0.07	0.07	0.07	0.07	0.07	0.07
**Subj 2**	0.02	0.07	0.04	0.07	0.07	0.06	0.06	0.06	0.06	0.06	0.06	0.07	0.08	0.08	0.08	0.08	0.08	0.07	0.07	0.07	0.07	0.07	0.07	0.07	0.07	0.07	0.07	0.07	0.07	0.07
**Subj 3**	0.05	0.03	0.04	0.03	0.03	0.03	0.01	0.02	0.02	0.02	0.03	0.03	0.02	0.01	0.02	0.01	0.02	0.02	0.02	0.02	0.02	0.02	0.01	0.01	0.01	0.00	0.00	0.00	0.00	0.00
**Subj 4**	0.44	0.36	0.34	0.30	0.32	0.33	0.32	0.34	0.33	0.34	0.33	0.33	0.33	0.34	0.35	0.34	0.34	0.34	0.35	0.34	0.34	0.34	0.33	0.34	0.33	0.33	0.34	0.33	0.34	0.33
**Subj 5**	0.24	0.27	0.26	0.27	0.29	0.32	0.33	0.29	0.31	0.32	0.32	0.33	0.33	0.36	0.37	0.36	0.36	0.35	0.36	0.35	0.36	0.36	0.35	0.35	0.35	0.35	0.35	0.35	0.35	0.35
**Subj 6**	0.04	0.01	0.01	0.00	0.01	0.00	0.01	0.01	0.01	0.01	0.02	0.02	0.02	0.01	0.01	0.02	0.02	0.02	0.01	0.01	0.01	0.02	0.02	0.02	0.02	0.02	0.02	0.02	0.02	0.02
**Subj 7**	0.03	0.03	0.05	0.05	0.05	0.06	0.06	0.07	0.06	0.05	0.06	0.06	0.05	0.05	0.05	0.05	0.04	0.05	0.05	0.04	0.04	0.04	0.04	0.04	0.04	0.04	0.05	0.05	0.05	0.06
**Subj 8**	0.09	0.01	0.02	0.18	0.13	0.18	0.23	0.17	0.20	0.24	0.26	0.28	0.29	0.29	0.31	0.31	0.31	0.31	0.33	0.34	0.34	0.35	0.34	0.33	0.33	0.30	0.30	0.30	0.28	0.27
**Subj 9**	0.01	0.01	0.01	0.01	0.00	0.01	0.01	0.02	0.04	0.06	0.06	0.06	0.05	0.04	0.05	0.04	0.04	0.04	0.03	0.03	0.03	0.02	0.02	0.00	0.01	0.01	0.00	0.00	0.00	0.00
**Subj 10**	0.19	0.11	0.09	0.07	0.04	0.01	0.01	0.00	0.00	0.01	0.01	0.00	0.00	0.00	0.00	0.01	0.01	0.02	0.05	0.05	0.05	0.05	0.05	0.05	0.05	0.05	0.05	0.05	0.05	0.04

CV DB DS Time ratio

	**1 trial**	**2 trials**	**3 trials**	**4 trials**	**5 trials**	**6 trials**	**7 trials**	**8 trials**	**9 trials**	**10 trials**	**11 trials**	**12 trials**	**13 trials**	**14 trials**	**15 trials**	**16 trials**	**17 trials**	**18 trials**	**19 trials**	**20 trials**	**21 trials**	**22 trials**	**23 trials**	**24 trials**	**25 trials**	**26 trials**	**27 trials**	**28 trials**	**29 trials**	**30 trials**
**Subj 1**	0.02	0.18	0.15	0.15	0.14	0.17	0.19	0.16	0.17	0.15	0.17	0.17	0.15	0.14	0.17	0.16	0.18	0.18	0.17	0.16	0.17	0.17	0.16	0.17	0.16	0.16	0.16	0.16	0.16	0.15
**Subj 2**	0.11	0.03	0.06	0.06	0.05	0.03	0.02	0.02	0.02	0.03	0.01	0.03	0.02	0.00	0.02	0.02	0.01	0.00	0.01	0.01	0.01	0.00	0.01	0.01	0.00	0.01	0.02	0.02	0.01	0.01
**Subj 3**	0.09	0.12	0.01	0.04	0.07	0.08	0.15	0.15	0.14	0.16	0.14	0.13	0.14	0.15	0.15	0.15	0.14	0.15	0.15	0.14	0.15	0.16	0.16	0.16	0.16	0.17	0.18	0.19	0.19	0.19
**Subj 4**	0.02	0.11	0.03	0.10	0.09	0.05	0.08	0.08	0.05	0.01	0.00	0.04	0.08	0.05	0.04	0.04	0.06	0.06	0.06	0.05	0.03	0.04	0.04	0.03	0.02	0.02	0.03	0.02	0.01	0.02
**Subj 5**	0.15	0.10	0.07	0.05	0.01	0.05	0.00	0.01	0.03	0.01	0.07	0.05	0.06	0.10	0.05	0.05	0.08	0.09	0.10	0.11	0.11	0.10	0.10	0.12	0.12	0.12	0.10	0.10	0.10	0.09
**Subj 6**	0.01	0.08	0.06	0.07	0.05	0.05	0.03	0.02	0.01	0.01	0.01	0.01	0.01	0.01	0.01	0.00	0.01	0.00	0.01	0.00	0.00	0.00	0.00	0.00	0.00	0.00	0.00	0.00	0.00	0.00
**Subj 7**	0.04	0.16	0.11	0.09	0.10	0.11	0.12	0.12	0.10	0.10	0.12	0.12	0.12	0.11	0.11	0.09	0.10	0.10	0.10	0.07	0.07	0.06	0.06	0.07	0.07	0.07	0.07	0.08	0.07	0.07
**Subj 8**	0.14	0.09	0.03	0.20	0.09	0.10	0.23	0.16	0.25	0.32	0.34	0.37	0.39	0.39	0.41	0.43	0.44	0.45	0.48	0.51	0.53	0.57	0.55	0.56	0.54	0.48	0.47	0.45	0.41	0.41
**Subj 9**	0.79	0.30	0.35	0.24	0.30	0.15	0.17	0.15	0.22	0.09	0.07	0.06	0.07	0.07	0.06	0.06	0.04	0.04	0.05	0.05	0.05	0.05	0.06	0.09	0.09	0.08	0.09	0.07	0.08	0.07
**Subj 10**	0.05	0.09	0.14	0.14	0.12	0.14	0.22	0.21	0.19	0.14	0.12	0.11	0.11	0.11	0.12	0.12	0.11	0.10	0.25	0.25	0.25	0.25	0.25	0.24	0.23	0.22	0.23	0.25	0.24	0.23

CV DS FT Time ratio

	**1 trial**	**2 trials**	**3 trials**	**4 trials**	**5 trials**	**6 trials**	**7 trials**	**8 trials**	**9 trials**	**10 trials**	**11 trials**	**12 trials**	**13 trials**	**14 trials**	**15 trials**	**16 trials**	**17 trials**	**18 trials**	**19 trials**	**20 trials**	**21 trials**	**22 trials**	**23 trials**	**24 trials**	**25 trials**	**26 trials**	**27 trials**	**28 trials**	**29 trials**	**30 trials**
**Subj 1**	0.50	0.36	0.13	0.15	0.09	0.23	0.28	0.23	0.28	0.27	0.20	0.20	0.13	0.12	0.11	0.10	0.10	0.25	0.30	0.37	0.37	0.36	0.38	0.37	0.38	0.36	0.38	0.36	0.35	0.36
**Subj 2**	0.01	0.07	0.01	0.15	0.16	0.14	0.10	0.05	0.01	0.01	0.02	0.00	0.01	0.01	0.01	0.01	0.01	0.00	0.01	0.01	0.02	0.00	0.00	0.02	0.03	0.03	0.04	0.02	0.03	0.02
**Subj 3**	0.60	0.34	0.23	0.22	0.07	0.05	0.01	0.01	0.03	0.01	0.05	0.06	0.01	0.02	0.04	0.02	0.01	0.02	0.02	0.02	0.01	0.02	0.03	0.02	0.02	0.00	0.01	0.00	0.01	0.01
**Subj 4**	0.15	0.17	0.23	0.22	0.20	0.14	0.28	0.24	0.22	0.19	0.22	0.19	0.17	0.17	0.19	0.18	0.21	0.20	0.18	0.18	0.19	0.18	0.20	0.19	0.20	0.19	0.20	0.24	0.24	0.24
**Subj 5**	0.32	0.29	0.32	0.21	0.24	0.24	0.20	0.79	0.72	0.70	0.67	0.63	0.61	0.56	0.55	0.53	0.51	0.43	0.42	0.40	0.39	0.38	0.38	0.49	0.48	0.47	0.46	0.46	0.10	0.10
**Subj 6**	0.02	0.06	0.10	0.10	0.05	0.06	0.06	0.06	0.04	0.04	0.02	0.02	0.04	0.03	0.03	0.03	0.03	0.05	0.06	0.07	0.07	0.07	0.07	0.07	0.07	0.06	0.06	0.05	0.05	0.04
**Subj 7**	0.02	0.05	0.09	0.05	0.07	0.08	0.00	0.04	0.09	0.11	0.12	0.10	0.11	0.12	0.13	0.14	0.14	0.13	0.15	0.18	0.17	0.17	0.15	0.18	0.18	0.17	0.17	0.17	0.17	0.16
**Subj 8**	0.19	0.15	0.00	0.03	0.16	0.04	0.08	0.07	0.03	0.03	0.05	0.05	0.06	0.06	0.05	0.05	0.04	0.04	0.03	0.02	0.01	0.05	0.06	0.07	0.06	0.06	0.06	0.03	0.04	0.04
**Subj 9**	0.09	0.10	0.11	0.12	0.11	0.05	0.08	0.09	0.18	0.17	0.16	0.12	0.08	0.07	0.08	0.07	0.08	0.08	0.07	0.06	0.05	0.04	0.03	0.03	0.04	0.02	0.00	0.01	0.01	0.02
**Subj 10**	0.51	0.34	0.30	0.20	0.02	0.01	0.00	0.06	0.05	0.01	0.01	0.02	0.01	0.01	0.00	0.00	0.01	0.00	0.00	0.00	0.01	0.01	0.00	0.07	0.06	0.06	0.04	0.03	0.01	0.00

CV Backswing peak (measured in m·s^{-2})

	**1 trial**	**2 trials**	**3 trials**	**4 trials**	**5 trials**	**6 trials**	**7 trials**	**8 trials**	**9 trials**	**10 trials**	**11 trials**	**12 trials**	**13 trials**	**14 trials**	**15 trials**	**16 trials**	**17 trials**	**18 trials**	**19 trials**	**20 trials**	**21 trials**	**22 trials**	**23 trials**	**24 trials**	**25 trials**	**26 trials**	**27 trials**	**28 trials**	**29 trials**	**30 trials**
**Subj 1**	0.04	0.01	0.04	0.04	0.04	0.04	0.05	0.04	0.03	0.00	0.01	0.03	0.03	0.04	0.05	0.04	0.04	0.04	0.03	0.02	0.03	0.02	0.02	0.02	0.02	0.01	0.00	0.00	0.00	0.00
**Subj 2**	0.41	0.13	0.07	0.05	0.00	0.03	0.03	0.04	0.05	0.06	0.06	0.04	0.02	0.00	0.01	0.01	0.01	0.01	0.02	0.04	0.05	0.04	0.03	0.03	0.04	0.02	0.02	0.02	0.03	0.03
**Subj 3**	0.05	0.10	0.10	0.11	0.11	0.12	0.14	0.14	0.14	0.12	0.10	0.10	0.10	0.09	0.09	0.08	0.08	0.08	0.08	0.08	0.07	0.07	0.07	0.07	0.06	0.06	0.06	0.07	0.07	0.07
**Subj 4**	0.23	0.15	0.08	0.00	0.02	0.07	0.08	0.10	0.09	0.07	0.08	0.07	0.06	0.04	0.03	0.04	0.05	0.07	0.06	0.08	0.08	0.09	0.08	0.08	0.07	0.08	0.08	0.08	0.08	0.10
**Subj 5**	0.46	0.18	0.24	0.14	0.08	0.06	0.08	0.07	0.11	0.11	0.15	0.12	0.11	0.07	0.06	0.05	0.07	0.13	0.15	0.15	0.16	0.17	0.18	0.19	0.18	0.17	0.17	0.18	0.18	0.18
**Subj 6**	0.02	0.15	0.04	0.04	0.03	0.05	0.05	0.06	0.08	0.09	0.09	0.06	0.06	0.06	0.06	0.07	0.06	0.06	0.07	0.07	0.07	0.07	0.06	0.06	0.06	0.06	0.06	0.06	0.06	0.06
**Subj 7**	0.12	0.08	0.01	0.01	0.02	0.02	0.03	0.05	0.06	0.05	0.04	0.04	0.03	0.02	0.02	0.01	0.00	0.01	0.02	0.01	0.01	0.01	0.01	0.01	0.02	0.02	0.02	0.02	0.01	0.00
**Subj 8**	0.10	0.06	0.21	0.12	0.21	0.16	0.18	0.17	0.16	0.18	0.16	0.14	0.13	0.13	0.10	0.11	0.10	0.11	0.11	0.11	0.11	0.09	0.10	0.10	0.09	0.10	0.09	0.10	0.10	0.10
**Subj 9**	0.29	0.01	0.14	0.08	0.06	0.04	0.03	0.05	0.14	0.12	0.08	0.05	0.05	0.07	0.03	0.02	0.01	0.01	0.01	0.01	0.00	0.01	0.02	0.01	0.02	0.02	0.06	0.08	0.06	0.07
**Subj 10**	0.27	0.20	0.22	0.16	0.13	0.15	0.23	0.23	0.24	0.19	0.21	0.22	0.22	0.23	0.21	0.19	0.20	0.19	0.23	0.23	0.23	0.21	0.20	0.20	0.19	0.19	0.19	0.21	0.22	0.22

CV Downswing peak (measured in m·s^{-2})

	**1 trial**	**2 trials**	**3 trials**	**4 trials**	**5 trials**	**6 trials**	**7 trials**	**8 trials**	**9 trials**	**10 trials**	**11 trials**	**12 trials**	**13 trials**	**14 trials**	**15 trials**	**16 trials**	**17 trials**	**18 trials**	**19 trials**	**20 trials**	**21 trials**	**22 trials**	**23 trials**	**24 trials**	**25 trials**	**26 trials**	**27 trials**	**28 trials**	**29 trials**	**30 trials**
**Subj 1**	0.04	0.01	0.02	0.01	0.05	0.05	0.04	0.04	0.03	0.03	0.03	0.03	0.03	0.02	0.01	0.00	0.00	0.01	0.00	0.00	0.00	0.01	0.01	0.01	0.02	0.01	0.01	0.01	0.01	0.02
**Subj 2**	0.17	0.17	0.16	0.12	0.16	0.12	0.09	0.08	0.06	0.09	0.09	0.10	0.09	0.08	0.07	0.07	0.07	0.08	0.08	0.06	0.07	0.08	0.07	0.07	0.06	0.07	0.06	0.06	0.05	0.06
**Subj 3**	0.15	0.10	0.10	0.07	0.05	0.02	0.02	0.02	0.02	0.01	0.01	0.01	0.00	0.00	0.00	0.01	0.01	0.01	0.01	0.01	0.01	0.02	0.01	0.01	0.00	0.00	0.00	0.00	0.00	0.01
**Subj 4**	0.22	0.16	0.08	0.12	0.11	0.10	0.07	0.05	0.05	0.05	0.04	0.05	0.05	0.05	0.05	0.05	0.05	0.04	0.05	0.05	0.05	0.06	0.06	0.05	0.06	0.06	0.05	0.05	0.05	0.05
**Subj 5**	0.10	0.01	0.18	0.17	0.09	0.06	0.05	0.17	0.16	0.14	0.12	0.12	0.11	0.10	0.08	0.06	0.06	0.06	0.05	0.05	0.04	0.04	0.05	0.03	0.03	0.03	0.03	0.02	0.06	0.06
**Subj 6**	0.09	0.08	0.03	0.05	0.01	0.02	0.01	0.01	0.01	0.02	0.01	0.01	0.00	0.01	0.01	0.01	0.00	0.00	0.00	0.00	0.01	0.01	0.01	0.01	0.01	0.00	0.00	0.00	0.00	0.00
**Subj 7**	0.04	0.04	0.07	0.07	0.04	0.02	0.00	0.00	0.00	0.03	0.00	0.01	0.01	0.03	0.03	0.04	0.04	0.04	0.04	0.03	0.02	0.01	0.01	0.01	0.01	0.00	0.02	0.02	0.01	0.01
**Subj 8**	0.25	0.16	0.06	0.09	0.11	0.11	0.10	0.09	0.08	0.07	0.07	0.07	0.06	0.05	0.05	0.04	0.03	0.03	0.02	0.01	0.02	0.02	0.02	0.03	0.03	0.03	0.04	0.03	0.03	0.04
**Subj 9**	0.05	0.11	0.08	0.07	0.09	0.06	0.05	0.07	0.06	0.06	0.05	0.06	0.06	0.07	0.06	0.06	0.06	0.06	0.06	0.06	0.05	0.06	0.06	0.06	0.06	0.06	0.07	0.07	0.07	0.06
**Subj 10**	0.22	0.25	0.21	0.17	0.13	0.11	0.09	0.11	0.12	0.11	0.11	0.12	0.12	0.12	0.12	0.11	0.10	0.09	0.07	0.07	0.07	0.07	0.08	0.07	0.07	0.06	0.06	0.06	0.07	0.08

CV Backswing peak time (measured in $m \cdot s^{-2}$)

	**1 trial**	**2 trials**	**3 trials**	**4 trials**	**5 trials**	**6 trials**	**7 trials**	**8 trials**	**9 trials**	**10 trials**	**11 trials**	**12 trials**	**13 trials**	**14 trials**	**15 trials**	**16 trials**	**17 trials**	**18 trials**	**19 trials**	**20 trials**	**21 trials**	**22 trials**	**23 trials**	**24 trials**	**25 trials**	**26 trials**	**27 trials**	**28 trials**	**29 trials**	**30 trials**
**Subj 1**	0.09	0.10	0.10	0.19	0.22	0.29	0.29	0.28	0.27	0.26	0.27	0.28	0.27	0.27	0.27	0.27	0.27	0.26	0.25	0.24	0.23	0.23	0.23	0.23	0.21	0.21	0.21	0.21	0.22	0.22
**Subj 2**	0.17	0.16	0.14	0.26	0.23	0.20	0.17	0.17	0.16	0.13	0.13	0.14	0.14	0.12	0.15	0.15	0.15	0.14	0.13	0.13	0.12	0.12	0.13	0.13	0.14	0.14	0.14	0.14	0.15	0.15
**Subj 3**	0.22	0.07	0.16	0.20	0.21	0.24	0.29	0.28	0.28	0.29	0.26	0.25	0.26	0.29	0.27	0.27	0.26	0.26	0.26	0.25	0.27	0.27	0.27	0.28	0.29	0.30	0.31	0.32	0.32	0.32
**Subj 4**	0.22	0.16	0.24	0.26	0.29	0.31	0.30	0.33	0.33	0.35	0.28	0.26	0.24	0.29	0.30	0.29	0.27	0.27	0.28	0.28	0.30	0.29	0.28	0.29	0.29	0.28	0.28	0.28	0.29	0.29
**Subj 5**	0.42	0.42	0.42	0.41	0.46	0.49	0.44	0.41	0.39	0.43	0.39	0.40	0.39	0.37	0.38	0.38	0.38	0.37	0.36	0.34	0.35	0.35	0.34	0.33	0.32	0.33	0.36	0.35	0.35	0.35
**Subj 6**	0.06	0.09	0.12	0.10	0.09	0.07	0.03	0.03	0.04	0.03	0.02	0.04	0.03	0.04	0.03	0.04	0.03	0.03	0.05	0.04	0.04	0.04	0.04	0.03	0.03	0.03	0.03	0.03	0.03	0.03
**Subj 7**	0.33	0.29	0.23	0.24	0.24	0.22	0.23	0.24	0.20	0.19	0.20	0.23	0.20	0.20	0.20	0.20	0.19	0.19	0.20	0.16	0.16	0.17	0.16	0.17	0.18	0.18	0.19	0.19	0.19	0.18
**Subj 8**	0.21	0.06	0.39	0.66	0.65	0.81	0.85	0.58	0.64	0.69	0.72	0.75	0.75	0.76	0.78	0.77	0.75	0.73	0.76	0.78	0.80	0.82	0.82	0.82	0.81	0.81	0.80	0.79	0.72	0.71
**Subj 9**	0.39	0.19	0.04	0.14	0.10	0.07	0.08	0.03	0.09	0.03	0.07	0.09	0.07	0.03	0.07	0.03	0.03	0.07	0.06	0.07	0.06	0.06	0.06	0.02	0.03	0.06	0.04	0.06	0.05	0.05
**Subj 10**	0.06	0.19	0.24	0.26	0.26	0.24	0.31	0.25	0.23	0.22	0.22	0.22	0.20	0.18	0.18	0.20	0.21	0.20	0.23	0.24	0.25	0.25	0.25	0.25	0.25	0.24	0.24	0.26	0.25	0.24

CV Downswing peak time (measured in $m \cdot s^{-2}$)

	**1 trial**	**2 trials**	**3 trials**	**4 trials**	**5 trials**	**6 trials**	**7 trials**	**8 trials**	**9 trials**	**10 trials**	**11 trials**	**12 trials**	**13 trials**	**14 trials**	**15 trials**	**16 trials**	**17 trials**	**18 trials**	**19 trials**	**20 trials**	**21 trials**	**22 trials**	**23 trials**	**24 trials**	**25 trials**	**26 trials**	**27 trials**	**28 trials**	**29 trials**	**30 trials**
**Subj 1**	0.02	0.03	0.02	0.01	0.00	0.00	0.00	0.03	0.02	0.03	0.02	0.02	0.04	0.04	0.03	0.02	0.00	0.00	0.01	0.01	0.00	0.00	0.01	0.00	0.00	0.00	0.00	0.00	0.01	0.01
**Subj 2**	0.02	0.07	0.08	0.07	0.11	0.09	0.12	0.13	0.12	0.12	0.12	0.14	0.14	0.15	0.15	0.15	0.16	0.15	0.14	0.14	0.14	0.13	0.13	0.12	0.12	0.13	0.12	0.13	0.12	0.12
**Subj 3**	0.09	0.15	0.21	0.28	0.33	0.32	0.31	0.28	0.29	0.29	0.29	0.26	0.25	0.26	0.27	0.27	0.29	0.27	0.27	0.28	0.28	0.29	0.28	0.27	0.26	0.27	0.27	0.28	0.28	0.29
**Subj 4**	0.31	0.19	0.22	0.21	0.15	0.16	0.18	0.20	0.23	0.27	0.25	0.29	0.32	0.29	0.29	0.29	0.30	0.29	0.31	0.30	0.30	0.29	0.30	0.30	0.29	0.28	0.27	0.27	0.28	0.29
**Subj 5**	0.31	0.41	0.27	0.24	0.27	0.24	0.25	0.18	0.22	0.20	0.25	0.25	0.25	0.34	0.35	0.36	0.38	0.35	0.34	0.32	0.33	0.34	0.32	0.34	0.32	0.32	0.33	0.33	0.33	0.33
**Subj 6**	0.05	0.07	0.05	0.02	0.00	0.00	0.01	0.01	0.01	0.02	0.00	0.03	0.03	0.03	0.02	0.03	0.03	0.03	0.03	0.03	0.03	0.03	0.03	0.03	0.02	0.02	0.02	0.02	0.02	0.02
**Subj 7**	0.04	0.06	0.01	0.04	0.03	0.03	0.04	0.03	0.03	0.08	0.03	0.02	0.01	0.01	0.02	0.06	0.05	0.05	0.06	0.07	0.07	0.08	0.08	0.06	0.06	0.06	0.06	0.07	0.07	0.07
**Subj 8**	0.02	0.06	0.07	0.07	0.27	0.13	0.11	0.09	0.07	0.06	0.06	0.06	0.07	0.07	0.08	0.07	0.06	0.06	0.05	0.05	0.01	0.02	0.02	0.04	0.03	0.03	0.02	0.02	0.02	0.01
**Subj 9**	0.29	0.22	0.16	0.15	0.18	0.10	0.13	0.12	0.20	0.17	0.14	0.13	0.14	0.14	0.13	0.14	0.12	0.11	0.11	0.09	0.08	0.08	0.07	0.07	0.07	0.07	0.04	0.04	0.05	0.04
**Subj 10**	0.13	0.04	0.07	0.11	0.10	0.06	0.06	0.03	0.08	0.00	0.00	0.00	0.01	0.01	0.02	0.01	0.02	0.00	0.03	0.02	0.02	0.03	0.03	0.03	0.04	0.04	0.05	0.06	0.05	0.05

CV Total resultant velocity (measured in $m \cdot s^{-2}$)

	**1 trial**	**2 trials**	**3 trials**	**4 trials**	**5 trials**	**6 trials**	**7 trials**	**8 trials**	**9 trials**	**10 trials**	**11 trials**	**12 trials**	**13 trials**	**14 trials**	**15 trials**	**16 trials**	**17 trials**	**18 trials**	**19 trials**	**20 trials**	**21 trials**	**22 trials**	**23 trials**	**24 trials**	**25 trials**	**26 trials**	**27 trials**	**28 trials**	**29 trials**	**30 trials**
**Subj 1**	0.00	0.00	0.12	0.10	0.05	0.12	0.14	0.13	0.13	0.11	0.09	0.10	0.09	0.09	0.09	0.07	0.06	0.08	0.10	0.11	0.11	0.10	0.12	0.11	0.12	0.12	0.12	0.12	0.12	0.12
**Subj 2**	0.10	0.11	0.08	0.13	0.12	0.10	0.09	0.07	0.06	0.06	0.07	0.07	0.07	0.07	0.06	0.07	0.07	0.06	0.06	0.06	0.07	0.07	0.06	0.06	0.06	0.06	0.06	0.06	0.05	0.05
**Subj 3**	0.30	0.21	0.17	0.13	0.09	0.05	0.04	0.04	0.04	0.04	0.05	0.06	0.04	0.04	0.04	0.03	0.03	0.04	0.05	0.05	0.05	0.04	0.05	0.05	0.05	0.05	0.05	0.04	0.05	0.04
**Subj 4**	0.50	0.46	0.41	0.38	0.38	0.39	0.37	0.36	0.35	0.36	0.37	0.37	0.36	0.36	0.36	0.36	0.37	0.37	0.37	0.37	0.37	0.38	0.38	0.37	0.37	0.37	0.38	0.38	0.38	0.38
**Subj 5**	0.38	0.36	0.20	0.22	0.26	0.33	0.35	0.32	0.36	0.36	0.36	0.37	0.37	0.38	0.37	0.37	0.39	0.36	0.38	0.38	0.38	0.39	0.36	0.36	0.36	0.36	0.38	0.38	0.38	0.39
**Subj 6**	0.06	0.06	0.00	0.02	0.01	0.00	0.01	0.02	0.04	0.05	0.04	0.03	0.03	0.02	0.02	0.02	0.02	0.02	0.02	0.02	0.01	0.01	0.01	0.01	0.01	0.01	0.01	0.01	0.01	0.01
**Subj 7**	0.03	0.01	0.01	0.02	0.01	0.01	0.01	0.04	0.06	0.06	0.06	0.06	0.06	0.07	0.07	0.07	0.07	0.06	0.07	0.07	0.06	0.05	0.04	0.05	0.05	0.04	0.03	0.04	0.04	0.04
**Subj 8**	0.05	0.00	0.03	0.00	0.05	0.04	0.04	0.05	0.03	0.01	0.02	0.01	0.00	0.00	0.01	0.02	0.02	0.02	0.02	0.03	0.04	0.05	0.04	0.03	0.03	0.03	0.03	0.03	0.03	0.03
**Subj 9**	0.04	0.09	0.09	0.11	0.09	0.08	0.05	0.06	0.05	0.05	0.03	0.04	0.05	0.05	0.04	0.04	0.05	0.05	0.05	0.06	0.05	0.06	0.06	0.06	0.06	0.06	0.07	0.07	0.07	0.06
**Subj 10**	0.05	0.08	0.08	0.04	0.00	0.03	0.02	0.04	0.05	0.05	0.06	0.06	0.06	0.05	0.05	0.05	0.05	0.05	0.08	0.08	0.07	0.07	0.08	0.07	0.07	0.06	0.07	0.06	0.07	0.07

CV Backswing resultant velocity (measured in $m \cdot s^{-2}$)

	**1 trial**	**2 trials**	**3 trials**	**4 trials**	**5 trials**	**6 trials**	**7 trials**	**8 trials**	**9 trials**	**10 trials**	**11 trials**	**12 trials**	**13 trials**	**14 trials**	**15 trials**	**16 trials**	**17 trials**	**18 trials**	**19 trials**	**20 trials**	**21 trials**	**22 trials**	**23 trials**	**24 trials**	**25 trials**	**26 trials**	**27 trials**	**28 trials**	**29 trials**	**30 trials**
**Subj 1**	0.19	0.26	0.24	0.21	0.21	0.22	0.22	0.19	0.20	0.18	0.16	0.15	0.15	0.14	0.14	0.14	0.14	0.14	0.15	0.15	0.14	0.14	0.14	0.14	0.14	0.14	0.14	0.14	0.14	0.13
**Subj 2**	0.14	0.02	0.01	0.04	0.02	0.03	0.02	0.02	0.03	0.04	0.05	0.03	0.01	0.01	0.01	0.02	0.03	0.03	0.04	0.05	0.06	0.06	0.06	0.06	0.06	0.05	0.05	0.05	0.05	0.05
**Subj 3**	0.19	0.09	0.07	0.03	0.00	0.00	0.05	0.05	0.05	0.05	0.04	0.03	0.04	0.03	0.03	0.02	0.01	0.01	0.01	0.01	0.01	0.02	0.01	0.01	0.01	0.01	0.01	0.02	0.02	0.03
**Subj 4**	0.72	0.62	0.47	0.38	0.39	0.43	0.42	0.46	0.43	0.40	0.42	0.39	0.37	0.37	0.37	0.37	0.35	0.36	0.36	0.38	0.39	0.38	0.37	0.38	0.37	0.37	0.38	0.37	0.38	0.38
**Subj 5**	0.47	0.39	0.40	0.35	0.33	0.37	0.37	0.33	0.38	0.37	0.39	0.39	0.39	0.34	0.34	0.33	0.34	0.38	0.40	0.40	0.41	0.42	0.42	0.41	0.40	0.40	0.41	0.42	0.42	0.42
**Subj 6**	0.05	0.11	0.02	0.01	0.03	0.03	0.03	0.04	0.05	0.06	0.05	0.02	0.03	0.02	0.03	0.02	0.02	0.02	0.02	0.03	0.03	0.03	0.02	0.02	0.01	0.01	0.01	0.01	0.01	0.02
**Subj 7**	0.04	0.03	0.02	0.00	0.01	0.02	0.04	0.05	0.05	0.05	0.05	0.05	0.03	0.03	0.03	0.03	0.03	0.03	0.03	0.00	0.00	0.00	0.00	0.01	0.01	0.01	0.01	0.02	0.02	0.03
**Subj 8**	0.19	0.12	0.13	0.05	0.20	0.07	0.02	0.08	0.00	0.04	0.07	0.12	0.14	0.16	0.20	0.20	0.20	0.20	0.22	0.23	0.26	0.29	0.26	0.26	0.26	0.21	0.21	0.19	0.15	0.15
**Subj 9**	0.71	0.26	0.41	0.25	0.31	0.13	0.17	0.20	0.28	0.24	0.21	0.18	0.17	0.19	0.16	0.14	0.12	0.08	0.10	0.09	0.09	0.08	0.08	0.11	0.10	0.09	0.08	0.04	0.06	0.05
**Subj 10**	0.07	0.00	0.02	0.00	0.01	0.08	0.16	0.15	0.16	0.11	0.12	0.12	0.12	0.12	0.13	0.12	0.13	0.12	0.37	0.36	0.35	0.34	0.32	0.31	0.30	0.29	0.29	0.31	0.31	0.30

CV Downswing resultant velocity (measured in $m \cdot s^{-2}$)

	**1 trial**	**2 trials**	**3 trials**	**4 trials**	**5 trials**	**6 trials**	**7 trials**	**8 trials**	**9 trials**	**10 trials**	**11 trials**	**12 trials**	**13 trials**	**14 trials**	**15 trials**	**16 trials**	**17 trials**	**18 trials**	**19 trials**	**20 trials**	**21 trials**	**22 trials**	**23 trials**	**24 trials**	**25 trials**	**26 trials**	**27 trials**	**28 trials**	**29 trials**	**30 trials**
**Subj 1**	0.00	0.05	0.02	0.02	0.05	0.04	0.04	0.04	0.03	0.04	0.05	0.04	0.04	0.03	0.03	0.03	0.03	0.03	0.03	0.03	0.03	0.03	0.03	0.03	0.03	0.03	0.03	0.03	0.03	0.03
**Subj 2**	0.10	0.06	0.04	0.03	0.04	0.03	0.02	0.02	0.02	0.02	0.02	0.03	0.02	0.02	0.01	0.02	0.02	0.02	0.01	0.01	0.02	0.02	0.01	0.01	0.01	0.01	0.01	0.01	0.01	0.01
**Subj 3**	0.10	0.00	0.05	0.04	0.03	0.03	0.06	0.06	0.06	0.06	0.06	0.07	0.06	0.06	0.05	0.04	0.04	0.05	0.05	0.04	0.04	0.04	0.04	0.05	0.05	0.05	0.05	0.05	0.06	0.05
**Subj 4**	0.63	0.45	0.39	0.38	0.38	0.40	0.34	0.34	0.34	0.36	0.34	0.35	0.36	0.36	0.36	0.36	0.36	0.37	0.37	0.36	0.35	0.36	0.36	0.35	0.35	0.35	0.36	0.35	0.35	0.35
**Subj 5**	0.40	0.37	0.26	0.26	0.29	0.32	0.35	0.24	0.28	0.28	0.30	0.31	0.32	0.34	0.33	0.34	0.35	0.35	0.37	0.37	0.38	0.38	0.37	0.39	0.38	0.39	0.38	0.39	0.37	0.37
**Subj 6**	0.08	0.06	0.01	0.02	0.00	0.00	0.00	0.00	0.02	0.03	0.02	0.00	0.01	0.00	0.01	0.00	0.01	0.01	0.01	0.01	0.01	0.01	0.00	0.00	0.00	0.00	0.00	0.00	0.00	0.00
**Subj 7**	0.02	0.05	0.08	0.07	0.06	0.03	0.03	0.03	0.03	0.03	0.03	0.03	0.02	0.02	0.02	0.02	0.02	0.02	0.02	0.01	0.02	0.01	0.01	0.02	0.01	0.01	0.00	0.01	0.01	0.01
**Subj 8**	0.13	0.04	0.02	0.04	0.01	0.05	0.06	0.06	0.07	0.06	0.06	0.06	0.05	0.05	0.04	0.03	0.04	0.04	0.03	0.03	0.03	0.04	0.04	0.04	0.04	0.04	0.04	0.03	0.03	0.03
**Subj 9**	0.09	0.15	0.16	0.14	0.13	0.08	0.08	0.09	0.10	0.09	0.08	0.09	0.09	0.09	0.09	0.08	0.07	0.08	0.08	0.08	0.08	0.07	0.07	0.08	0.09	0.08	0.09	0.08	0.08	0.08
**Subj 10**	0.03	0.07	0.06	0.07	0.06	0.08	0.04	0.04	0.05	0.08	0.09	0.09	0.09	0.09	0.09	0.08	0.08	0.08	0.06	0.05	0.05	0.06	0.06	0.06	0.06	0.06	0.06	0.05	0.05	0.05
